# Editorial: Transitional circulation

**DOI:** 10.3389/fped.2023.1328201

**Published:** 2023-11-09

**Authors:** Laura Mihaela Suciu, Stuart Brian Hooper, Patrick J. McNamara

**Affiliations:** ^1^ Department of Neonatology, University of Medicine Pharmacy Science and Technology George Emil Palade of Târgu Mures, Târgu Mures, Romania; ^2^ Department of Obstetrics and Gynecology, Hudson Institute for Medical Research, Monash University, Melbourne, VIC, Australia; ^3^ Department of Pediatrics, University of Iowa Stead Family Children’s Hospital, Iowa City, IA, United States

**Keywords:** hemodynamic evaluation, normative echocardiography data, abnormal transition, targeted neonatal echocardiography, cardiovascular monitoring

**Editorial on the Research Topic** Transitional circulation

The transition to extrauterine life represents one of the most challenging and vulnerable processes for the human body, thereby placing the newly born at risk of cardiopulmonary maladaptation. Identifying the etiology of disruption in the cardiocirculatory transition is difficult and often multifactorial. The collection of articles in this edition primarily focuses on the complexity of the transition, relevant physiological concepts and the value of enhanced neonatal cardiovascular monitoring. Failure of fetal-to-neonatal cardiopulmonary transition at birth can occur at multiple levels, commencing with an inability to aerate the lung. Although this is the primary stimulus for the decrease in pulmonary vascular resistance (PVR) at birth, if this fails or in instances of severe lung hypoplasia, both pulmonary blood flow and left ventricular preload decrease. Comprehensive knowledge of cardiopulmonary physiology and the unique intrinsic responses of the immature myocardium to preload (Frank-Starling law), afterload (stress-velocity relationship) and heart rate (force-frequency relationship) are essential to understanding normal vs. abnormal postnatal adaptation.

The fetal vascular shunts (duct arteriosus and foramen ovale) may play a differential role according to the underlying physiology. In patients with acute pulmonary hypertension and elevated right ventricular afterload (*right heart, abnormal transition)*, closure of the patent ductus arteriosus (DA) may lead to right ventricular dysfunction due to systolic wall stress and increased myocardial oxygen demand and pulmonary hypoperfusion. The consequences of abnormal transition to *the left heart* include poor left ventricular (LV) filling and low LV output which, despite normal LV systolic performance, reduces the flow of oxygenated blood into the systemic circulation. Persistent hypoxemia can lead to the redistribution of cardiac output, which decreases further the perfusion of some organs and thereby accelerates acidosis and cyanosis. In certain disease states*, abnormal transition* is associated with persistence of the fetal shunts. On the one hand, sustained right-to-left shunting at the DA and FO may lead to worsening of the hypoxemia. On the contrary, when PVR falls rapidly in extremely preterm babies progressive left-to-right DA shunting may lead to hypotension, pulmonary hemorrhage and intraventricular hemorrhage.

Deshpande et al. provide a comprehensive overview (Deshpande et al.) of current trends of hemodynamic evaluation in NICU during the transitional period in extremely low gestational age neonates (ELGANs). The newer modalities of cardiovascular monitoring such as targeted neonatal echocardiography (TnECHO), prefrontal cerebral near-infrared spectroscopy (NIRS) and cerebral saturation (CrSO2) were tested against the conventional methods of systolic and diastolic arterial blood pressure monitoring obtained by oscillometry. Simultaneous multimodal monitoring with TnECHO, NIRS, and CrSO2 may provide an enhanced understanding of the relationship between cerebral oxygenation and systemic blood flow during transition among vulnerable population of ELGANs. Of note, TnECHO enables bedside assessment of systemic and pulmonary blood flow, cardiac function and the presence and direction of the fetal vascular shunts (1). Thus, it enables enhanced diagnostic precision and earlier recognition of the underlying hemodynamic status and its impact both on health and disease state. Artificial intelligence and predictive analytics may facilitate the creation of algorithms to identify patients at greatest risk of neonatal morbidity where earlier intervention may be meritorious.

Characterization of the transition in at-risk populations has received little scientific attention. In the study by Suciu et al., TnECHO was used to compare echocardiography indices between small and appropriate for gestational age infants. The authors detected lower RV function and higher pulmonary vasculature resistance among intrauterine growth restricted (IUGR) infants during the transitional period (Suciu et al.). While these data highlight the differences between populations, the application of echocardiography and a comprehensive integrated multimodal approach for assessing the cardiovascular system has many limitations. First, a metareview of the literature related to characterization of the neonatal transition reveals that there are major gaps in normative echocardiography data; specifically, differences in timing of evaluation, training, and imaging techniques limit the ability to generate reliable conclusions (Suciu et al.). *Second*, longitudinal normative data in preterm babies during the early transitional period are lacking. There is almost no normative data in preterm infants born less than 24 weeks gestation. *Third*, differences in adaptive patterns according to sex, race or ethnicity are lacking. There is an urgent need to establish standardized echocardiography measurement techniques, protocols and training to advance our ability to recognize and intervene in the care of a baby with an abnormal transition. In addition, a high degree of methodological rigor in both image acquisition and measurement analyses are essential pre-requisites to enhance the generalizability and the validity of future research studies. The use of centralized system for echo review and analysis (CORE labs) for multicenter studies to (i) oversee echocardiography training, (ii) ensure quality assurance, and (iii) conduct all echocardiography analysis to ensure measurement consistency may further enhance the quality of all neonatal echocardiography research.
